# Uniting for greater impact: The crucial role of oncology nurses in cancer care

**DOI:** 10.1016/j.apjon.2023.100370

**Published:** 2023-12-27

**Authors:** Tateaki Naito

**Affiliations:** Division of Thoracic Oncology, Shizuoka Cancer Center, Shizuoka, Japan

As medical advances extend individual lifespans, more people are living with cancer and are emphasizing the importance of quality of life in their cancer journey. In this evolving landscape, the role of the oncology nurse is critical to unite diverse healthcare professionals and ensure cohesive, patient-centered care. *Asia–Pacific Journal of Oncology Nursing* (APJON) has seen an increase in submissions on multidisciplinary care based on advances in cancer therapeutics, supportive care, and digital health. Recent articles in *Asia–Pacific Journal of Oncology Nursing* have demonstrated the importance of nurse-led multidisciplinary care in managing symptoms or adverse events, nutritional status, and improving quality of life in head and neck,^1^ lung,^2^ esophageal,^3^ gynecologic,^4^ and pediatric cancers.^5^ This editorial focuses on the potential role and impact of oncology nurses in multidisciplinary care.

Integrating care from multiple professions can be challenging. This is especially true for complex oncologic conditions, such as cancer cachexia, where multiple oncology or supportive care services are provided, as shown in Fig. 1. Although each specialist brings sufficient expertise to the table, poor interprofessional communication often fragments the overall treatment plan. In addition, caregivers and the home healthcare team are not brought into the loop until the later stages of care. An unconnected circle of care leaves patients confused and disoriented. This scenario, described as a ‘bankrupt shuttlecock,’ emphasizes the need for a central figure to coordinate care.^6^ The oncology nurse can play three important roles in this coordination.

**Fig. 1:**
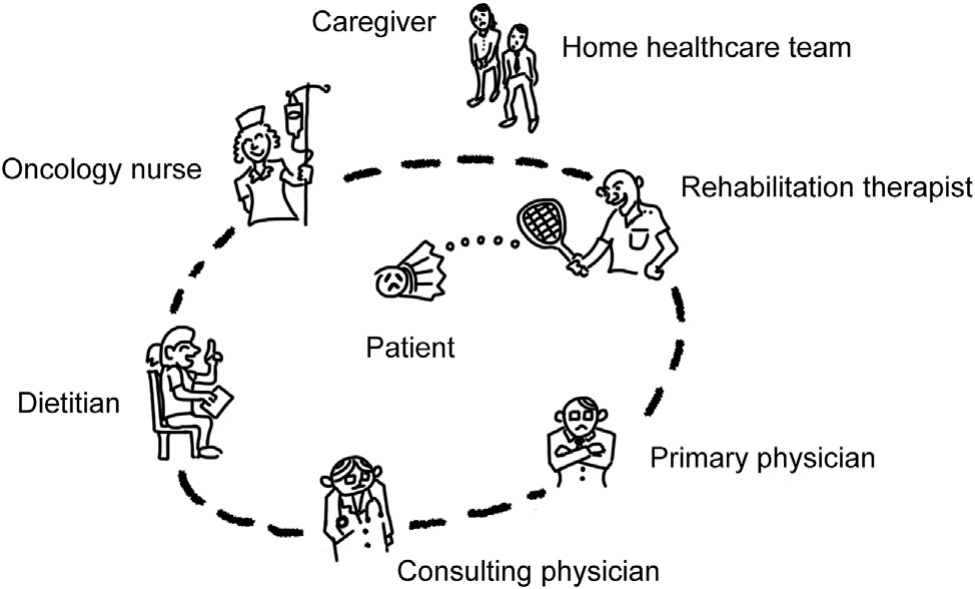
Pitfall of multidisciplinary cancer care. The figure was cited with modification from reference 11.

## Oncology nurse as central hub

Collaboration between healthcare professionals such as oncologists, physiotherapists, dietitians, palliative care specialists, pharmacists, and nurses is essential in the care of cachectic patients with advanced cancer receiving chemotherapy.^7^ Oncologists are faced with the difficult task of managing the growing body of evidence on cancer treatment while keeping-up with rapidly changing standards of care and insurance coverage. Due to their busy schedules, oncologists often have limited time to listen to the concerns of patients and caregivers, making it difficult to facilitate in-depth multidisciplinary collaboration. Oncology nurses are at the forefront of cancer care and can act as the hub of the multidisciplinary wheel.^8^ They ensure a unified approach to treatment by coordinating the efforts of different specialists and addressing the holistic needs of patients.

## Beyond clinical care: Advocacy and empathy

Oncology nurses are uniquely positioned to advocate for the needs and preferences of patients and caregivers, to understand their unspoken concerns, and to ensure that these are represented in care decisions. Behavior-change techniques can be used to educate patients and caregivers to promote self-care for cancer-related symptoms and treatment-related adverse events and to encourage the development of coping skills.^9^ This approach empowers patients and caregivers to effectively manage their condition, further emphasizing the critical role of oncology nurses in supporting holistic patient care.

## Communicating effectively: Bridging the gaps

**Fig. 2:**
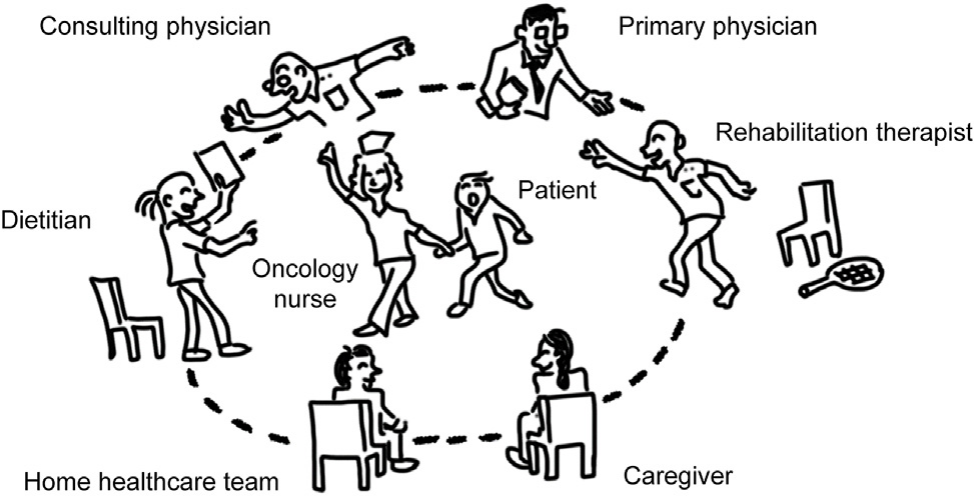
Hub role of oncology nurses in multidisciplinary cancer care. Legend: the figure was cited with modification from reference 11.

In the multidisciplinary care setting, patients and caregivers are often faced with an overwhelming amount of information. Within the constraints of short medical consultation times, they are asked to assess risks and make treatment decisions in the face of significant uncertainty.^10^ Oncology nurses can facilitate essential communication between patients and the healthcare team, as shown in Fig. 2. They translate complex medical information and ensure that patients are informed and are involved in their care decisions, reflecting their values and preferences.

In summary, the role of oncology nurses as facilitators is essential to address the identified pitfalls of multidisciplinary cancer care. They are the linchpin that facilitates dialog and collaboration among healthcare providers and ensures that all members of the care team are working toward a common goal. This collaborative unity is essential to the delivery of high-quality, patient-centered cancer care. To paraphrase an African proverb, ‘If you want to go fast, go alone. If you want to go far, go together,’ their collaborative efforts are key to improving not only the length but also the quality of the patient journey. It truly embodies the spirit of going far together in cancer care.

## Funding

This work was supported by the 10.13039/100009619Japan Agency for Medical Research and Development (AMED) under Grant No. 21ck0106673h0001. The funders had no role in considering the study design or in the collection, analysis, interpretation of data, writing of the report, or decision to submit the article for publication.

## Ethics statement

Not required.

## Declaration of competing interests

The author received a lecture fee from ONO Pharmaceutical CO. Ltd and research funding from Otsuka Pharmaceutical CO. Ltd.

## Declaration of Generative AI and AI-assisted technologies in the writing process

No AI tools/services were used during the preparation of this work.
